# Dangers of Santonin

**Published:** 1887-04

**Authors:** 


### *DANGERS OF SANTONIN.*

Dr. Laure, at a recent meeting of the Lyons Medical Society, related an interesting case of santonin-poisoning. On December 25th last, he was called to visit a child three and a-half years old. The parents attributed the evident intoxication to some black lead which the child had daubed over his lips. As black lead, notwithstanding its name, consists of nearly pure carbon and contains no lead, the case was somewhat obscure. The patient was lying on his back, in a state of deep prostration, now and then interrupted by sharp cries. The child then would bend up its knees and place its hands on its abdomen, apparently the seat of the pain. This fit over, he would fall again into the former somnolence. The face was of a livid paleness, the pupils dilated, breathing frequent, the pulse rapid and irregular, while the rectal temperature remained below 37° C. (98.6° F.) The stomach would immediately reject anything ingested.

Matters passed by the patient after the administration of a purgative enema, advised by the family pharmacist, showed nothing abnormal, excepting the deepened coloration of the liquid portions, and this was at first attributed to the drugs themselves from which the enema was composed.

On the whole, the symptoms were very perplexing, when the mother, on being interrogated again, remembered that *two days before* she had given the child a dose of ten centigrammes (1½ grains) of santonin. She even had a second powder like it, but, fortunately, did not administer it. It appeared, also, that the day before calling in the physician, the child had passed what the parents thought to be bloody urine. This color, Dr. Laure thinks, was simply due to santonin, as Wood states it to be the case finally.

Perhaps the worst symptom was the complete retention of urine, none having been passed within twenty-four hours. But once the case was clearly understood, the treatment was easy and the recovery rapid. A warm bath and a laxative enema restored the flow of urine, and a copious passage relieved the abdominal tympanitis. In a few days, the patient was well again.

This custom of dosing children with santonin seems to be a common one with the French working classes. As soon as the little ones have the slightest diarrhœa caused by teething, some parents conclude they have worms, and give them the medicine without any medical advice. Besides, they administer it simply powdered, without adjuvants or correctives.

As regards the use of santonin, Dr. Laure thinks the rule laid down by Benzinger, viz., as many grains as the child has years, four days in succession, is one that should be regarded with suspicion. He prefers Wood's advice — not to exceed one grain for a child less than two years.* Dr. Laure is also of opinion that it is very desirable to combine santonin with a purgative; calomel, for instance. As to the antidotes of santonin, ether and especially chloral have been recommended as the best by Becker and Binz. In a case when convulsions or nervous troubles occur, they would have been used willingly. But in the case just narrated, it was thought more advisable to facilitate the elimination of the poison by the kidneys and bowels, and afterwards to administer tonics.—*Paris Letter to Therapeutic Gazette.*

* It must be borne in mind that when modern French writers use the term "grain," which, fortunately, they seldom do, they mean five centigrammes, while the English grain is equal to about six and one-half centigrammes.

